# Sex-Specific Association between Systolic Blood Pressure Time in Target Range and Cardiovascular Outcomes: A Post-Hoc Analysis of the SPRINT Trial

**DOI:** 10.31083/RCM26262

**Published:** 2025-03-18

**Authors:** Yuekun Zhang, Wen Zheng, Chao Jiang, Wen Hao, Wei Gong, Yan Yan, Xiao Wang, Changsheng Ma, Shaoping Nie

**Affiliations:** ^1^Division of Cardiology, Beijing Anzhen Hospital, Capital Medical University, 100029 Beijing, China; ^2^Cardiometabolic Medicine Center, Fuwai Hospital, National Center for Cardiovascular Diseases, Chinese Academy of Medical Sciences and Peking Union Medical College, 100037 Beijing, China

**Keywords:** hypertension, systolic blood pressure, sex differences, time in target range

## Abstract

**Background:**

Systolic blood pressure time in target range (SBP TTR) is a novel metric for blood pressure control. Previous studies have demonstrated an inverse association between SBP TTR and risks of cardiovascular events, but sex differences have never been reported. This study aims to investigate the sex-specific differences in the relationship using data from the Systolic Blood Pressure Intervention Trial (SPRINT).

**Methods:**

This post hoc analysis included 8822 SPRINT participants with at least three follow-up systolic blood pressure (SBP) measurements within the first three months. SBP TTR was calculated using the Rosendaal method of linear interpolation. The primary endpoint was major adverse cardiovascular and cerebrovascular events (MACCE). Cox proportional hazards models and restricted cubic splines (RCS) were used to assess the association between SBP TTR and cardiovascular events.

**Results:**

Women accounted for 35.3% with a mean age of 68.6 ± 9.5 years, having a higher body mass index (*p* = 0.007) and a lower SBP TTR compared to men (*p* < 0.001). In the overall population and in women, each standard deviation (SD) increase in SBP TTR was associated with a reduced risk of MACCE (adjusted hazard ratio (HR) 0.89; 95% confidence interval (CI) 0.82–0.97; *p* = 0.007, and adjusted HR 0.85; 95% CI 0.74–0.99; *p* = 0.039, respectively) and acute decompensated heart failure (adjusted HR 0.86; 95% CI 0.73–0.99; *p* = 0.047, and adjusted HR 0.68; 95% CI 0.51–0.92; *p* = 0.011, respectively), while this was not observed in men. RCS indicated a similar trend in men only when SBP TTR exceeded 39%. Additional adjustments for mean SBP and SBP variability yielded similar outcomes.

**Conclusions:**

The study demonstrates that in women, a higher SBP TTR is associated with a reduced risk of MACCE and acute decompensated heart failure, while in men, a similar trend is observed only when SBP TTR is higher, underscoring the necessity of considering sex differences in personalized blood pressure management strategies.

**Clinical Trial Registration:**

NCT01206062, https://www.clinicaltrials.gov/expert-search?term=NCT01206062.

## 1. Introduction

Cardiovascular disease (CVD) remains the leading cause of mortality worldwide, with hypertension identified as a central and modifiable risk factor [1]. Effective management of hypertension is essential to reduce cardiovascular risk [2]. While traditional measures of blood pressure control do not account for variability over time or the overall quality of blood pressure management, systolic blood pressure time in target range (SBP TTR) has emerged as a novel measure that incorporates average blood pressure levels, stability, and variability [3, 4]. SBP TTR, defined as the percentage of time during which systolic blood pressure is within a specified range, has been shown to be associated with both cardiovascular events and all-cause mortality in hypertensive individuals, highlighting its potential utility in predicting cardiovascular risk [5, 6].

The effect of sex differences on this association has not been thoroughly investigated, despite the evidence linking SBP TTR to cardiovascular outcomes. This is particularly important given the known differences between women and men in blood pressure regulation and cardiovascular risk [7]. Women, particularly in the postmenopausal period, face unique challenges in blood pressure management due to hormonal changes that can lead to increased arterial stiffness, endothelial dysfunction, and unfavorable lipid distribution [8, 9, 10]. These factors may contribute to a higher cardiovascular risk in older women compared to their male counterparts [11].

Given the potential sex differences in the relationship between SBP TTR and cardiovascular risk, it is crucial to investigate this association in a sex-stratified manner, which will enable the development of more precise and personalized management strategies to improve patient prognosis [12]. To investigate this issue, we conducted a post hoc analysis of data from the Systolic Blood Pressure Intervention Trial (SPRINT), a multicenter, randomized, controlled trial of individuals at high risk for cardiovascular events [13]. We sought to provide insight into the sex-specific relationship between SBP TTR and cardiovascular outcomes.

## 2. Materials and Methods

### 2.1 Data Availability

All datasets and materials relevant to this study have been made publicly accessible via the BioLINCC data retrieved from https://biolincc.nhlbi.nih.gov/home/.

### 2.2 Study Design and Population

SPRINT was a multicenter, open-label, randomized controlled trial designed for individuals at increased cardiovascular risk, excluding those with a documented history of diabetes mellitus or stroke. The trial sought to compare the effectiveness of an aggressive systolic blood pressure management strategy (<120 mmHg) against a conventional approach (<140 mmHg) (NCT01206062). Participants received medications proven to reduce cardiovascular event risk, using the Adherence Scale to assess the medication adherence, along with recommendations for lifestyle modifications, including the Dietary Approaches to Stop Hypertension (DASH) diet and increased physical activity. The protocol and findings have been previously documented [14]. Authorization was secured from the respective institutional review boards, and all participants provided informed consent. This analysis included participants who underwent a minimum of three follow-up measurements within the initial three months after the baseline blood pressure measurement.

### 2.3 Blood Pressure (BP) Measurement and Estimation of SBP TTR

BP was measured by trained clinic staff. Values were obtained by averaging three seated measurements and were routinely measured at baseline, monthly for the initial three months, and every three months thereafter. SBP TTR is delineated as the proportion of time during the observation period wherein SBP values remain within the predefined target range. Systolic blood pressure (SBP) values throughout the observation period were derived using the linear interpolation method of Rosendaal which assumes a linear change between consecutive measurements to estimate SBP at each time point [15, 16]. As previously documented, the target range for the intensive group was defined as 110–130 mmHg with the upper limit aligning with current guideline recommendations, and the target range for the standard group was set at 120–140 mmHg [17, 18]. Ultimately, the percentage of time within this target range is calculated as the metric of SBP TTR. The calculation of SBP TTR, mean SBP, and SBP variability were based on all SBP data within the initial three months of the study.

### 2.4 Follow-up and Outcomes

Structured interviews were conducted quarterly with participants to ascertain the occurrence of potential events. The primary endpoint was major adverse cardiovascular and cerebrovascular events (MACCE), comprising stroke, non-fatal myocardial infarction, non-myocardial infarction acute coronary syndrome, cardiac death, and acute decompensated heart failure. Secondary endpoints encompassed the discrete constituents of MACCE.

### 2.5 Statistical Analysis

Quantitative data following a normal distribution were characterized by the mean ± SD, and differences were assessed through an independent sample *t*-tests or one-way analysis of variance. Non-normally distributed quantitative data were described using the median (interquartile range) and analyzed for disparities utilizing the Mann-Whitney U test or Kruskal-Wallis test. Qualitative data were delineated by frequencies and percentages, and chi-square test was applied to identify disparities across groups. Cox proportional hazards model was utilized to delineate the association between SBP TTR and cardiovascular events. Two models were applied in this study consistent with previously published literature: (1) a unadjusted model; (2) an adjusted model, accounting for the treatment group, age, race, a 10-year cardiovascular risk score, baseline SBP, body mass index (BMI), and renal insufficiency. Restricted cubic splines (RCS) with 3 knots at the 10th, 50th, and 90th percentiles of TTR were utilized to further explore the relationship between SBP TTR and cardiovascular events, based on the adjusted Cox model. Statistical analyses were conducted utilizing SPSS (version 27.0, IBM Corp, Armonk, NY, USA) and R software (version 4.3.2, R Foundation for Statistical Computing, Vienna, Austria), with a two-sided *p*-value < 0.05 regarded as statistically significant.

## 3. Results

### 3.1 Baseline Characteristics and Antihypertensive Interventions

8822 patients were included in the final analysis, with the exclusion of 539 participants due to the absence of a minimum of three follow-up SBP measurements within the initial three months or incomplete covariate information.

In the overall population, the mean age was 67.9 ± 9.4 years, with a median SBP TTR of 38% (14%–64%). In contrast to men, women, who accounted for 35.3% of the overall population, demonstrated a greater mean age (68.6 ± 9.5 vs 67.6 ± 9.3, *p* < 0.001), an elevated BMI (30.1 ± 6.5 vs 29.7 ± 5.1, *p* = 0.007), and a lower SBP TTR within the initial three months (37% (14%–61%) vs 39% (16%–67%), *p* < 0.001). No statistically significant difference was observed in the mean SBP between the two groups during the initial three months (129.5 ± 13.0 vs 129.8 ± 11.5, *p* = 0.241). Detailed clinical characteristics of the patients, stratified by sex or jointly by sex and SBP TTR, are available in Table 1 and **Supplementary Table 1**.

**Table 1.** **Baseline clinical characteristics by sex and SBP time in target range (TTR) categories**.

	Women (n = 3114)					Men (n = 5708)				
	TTR	TTR	TTR	TTR	*p* value	TTR	TTR	TTR	TTR	*p* value
	0% to <14%	14% to <38%	38% to <64%	64% to 100%		0% to <14%	14% to <38%	38% to <64%	64% to 100%	
	(n = 762)	(n = 876)	(n = 774)	(n = 702)		(n = 1368)	(n = 1462)	(n = 1343)	(n = 1535)	
Intensive arms	210 (27.6)	464 (53.0)	465 (60.1)	437 (62.3)	<0.001	288 (21.1)	779 (53.3)	773 (57.6)	1005 (65.5)	<0.001
Age, years	69.2 ± 9.7	68.5 ± 9.8	68.4 ± 9.4	68.3 ± 9.0	0.275	67.5 ± 9.3	68.0 ± 9.4	67.8 ± 9.2	67.1 ± 9.1	0.048
BMI, kg/m^2^	29.5 ± 6.4	30.1 ± 6.6	30.2 ± 6.5	30.6 ± 6.4	0.016	29.6 ± 5.2	29.7 ± 5.2	29.8 ± 5.1	29.8 ± 5.0	0.663
White	427 (56.0)	486 (55.5)	430 (55.6)	400 (57.0)	0.447	937 (68.5)	1043 (71.3)	985 (73.3)	1105 (72.0)	0.010
Current smoking	97 (12.7)	104 (11.9)	103 (13.3)	91 (13.0)	0.839	187 (13.7)	182 (12.4)	187 (13.9)	184 (12.0)	0.342
History of CVD	105 (13.8)	140 (16.0)	123 (15.9)	106 (15.1)	0.594	302 (22.1)	366 (25.0)	321 (23.9)	308 (20.1)	0.007
10-y ASCVD risk	17 (16–19)	17 (16–18)	17 (16–18)	17 (16–18)	<0.001	18 (16–20)	18 (16–19)	18 (16–19)	17 (15–19)	<0.001
Renal insufficiency^1^	247 (32.4)	275 (31.4)	241 (31.1)	223 (31.8)	0.954	316 (23.1)	377 (25.8)	343 (25.5)	347 (22.6)	0.096
Baseline SBP, mmHg	149.8 ± 12.2	147.7 ± 11.4	146.4 ± 11.0	145.1 ± 10.9	<0.001	146.6 ± 11.5	144.6 ± 11.3	143.0 ± 10.3	141.6 ± 9.3	<0.001
Baseline DBP, mmHg	80.0 ± 12.3	80.0 ± 12.2	79.9 ± 11.7	79.7 ± 11.3	0.961	81.2 ± 11.7	80.7 ± 11.6	80.1 ± 11.2	79.7 ± 10.8	0.002
TTR, %	0 (0–6)	26 (20–32)	50 (44–57)	80 (71–92)	<0.001	0 (0–4)	27 (21–32)	51 (46–57)	82 (72–96)	<0.001

Data are presented as mean ± SD, median (interquartile range), or n (%). ASCVD, arteriosclerotic cardiovascular disease; BMI, body mass index; CVD, cardiovascular disease; DBP, diastolic blood pressure; eGFR, glomerular filtration rate; SBP, systolic blood pressure; SD, standard deviation. ^1^eGFR <60 mL/min/1.73 m^2^.

The antihypertensive intervention strategies, stratified by the number of antihypertensive agents, showed no statistically significant differences between men and women, either in total or when categorized by SBP TTR quartile of the overall population. Notably, in patients who were administered a single type of antihypertensive agent, women exhibited a lower SBP TTR compared with men (40% ± 30% vs 42% ± 32%, *p* = 0.054) despite statistical non-significance. Additionally, the SBP TTR for women was significantly lower than that for men among patients receiving two (*p* < 0.001) or more medications (*p* = 0.016). Additional details are presented in** Supplementary Tables 2,3**.

### 3.2 Effect of Systolic Blood Pressure Time in Target Range on Prognosis

There were 673 MACCE in the overall population after a follow-up period of 3.8 (3.3–4.4) years (**Supplementary Table 4**). The incidence rate of MACCE in the overall population was 20.7 per 1000 person-years (95% confidence interval [CI], 19.1–22.3), which was 17.4 per 1000 person-years (95% CI, 15.0–20.0) for women and 22.5 per 1000 person-years (95% CI, 20.5–24.6) for men (Table 2).

**Table 2.** **Association between SBP time in target range (TTR) and risk of cardiovascular events**.

		IR^1^ (95% CI)	Unadjusted		Adjusted^3^	
			HR^2^ (95% CI)	*p* value	HR^2^ (95% CI)	*p* value
Major adverse cardiovascular and cerebrovascular events						
	Overall	20.7 (19.1–22.3)	0.84 (0.78–0.91)	<0.001	0.89 (0.82–0.97)	0.007
	Women	17.4 (15.0–20.0)	0.83 (0.72–0.96)	0.013	0.85 (0.74–0.99)	0.039
	Men	22.5 (20.5–24.6)	0.84 (0.77–0.92)	<0.001	0.91 (0.83–1.01)	0.071
Nonfatal myocardial infarction						
	Overall	7.8 (6.9–8.8)	0.84 (0.74–0.95)	0.007	0.91 (0.80–1.04)	0.167
	Women	6.6 (5.1–8.2)	0.82 (0.65–1.04)	0.103	0.90 (0.70–1.14)	0.373
	Men	8.5 (7.3–9.8)	0.84 (0.72–0.98)	0.024	0.91 (0.78–1.07)	0.270
Non-MI ACS						
	Overall	2.5 (2.0–3.1)	0.97 (0.78–1.20)	0.785	0.95 (0.76–1.20)	0.693
	Women	1.3 (0.8–2.2)	1.24 (0.77–1.99)	0.380	1.27 (0.77–2.07)	0.348
	Men	3.1 (2.4–4.0)	0.90 (0.70–1.14)	0.381	0.89 (0.69–1.16)	0.407
Stroke						
	Overall	4.8 (4.1–5.6)	0.71 (0.60–0.83)	<0.001	0.73 (0.61–0.88)	0.001
	Women	4.9 (3.7–6.4)	0.71 (0.54–0.94)	0.017	0.69 (0.51–0.92)	0.013
	Men	4.7 (3.8–5.7)	0.70 (0.57–0.87)	0.001	0.77 (0.62–0.97)	0.028
Acute decompensated heart failure						
	Overall	6.0 (5.1–6.8)	0.81 (0.70–0.94)	0.005	0.86 (0.73–0.99)	0.047
	Women	5.0 (3.8–6.4)	0.67 (0.51–0.89)	0.005	0.68 (0.51–0.92)	0.011
	Men	6.5 (5.4–7.6)	0.87 (0.73–1.03)	0.100	0.94 (0.78–1.13)	0.526
Cardiovascular death						
	Overall	3.8 (3.1–4.5)	0.86 (0.72–1.03)	0.102	0.94 (0.78–1.13)	0.517
	Women	2.9 (2.0–4.0)	1.04 (0.75–1.45)	0.809	1.16 (0.82–1.65)	0.391
	Men	4.3 (3.4–5.2)	0.79 (0.64–0.98)	0.032	0.88 (0.70–1.11)	0.277

ACS, acute coronary syndrome; HR, hazard ratio; IR, incidence rate; MI, myocardial infarction; SD, standard deviation; SBP, systolic blood pressure; CI, confidence interval. ^1^IR per 1000 person-years; ^2^HR per one SD increase in time in target range; ^3^adjusted for age, race, treatment group, 10-year cardiovascular risk score, baseline SBP, body mass index, and renal insufficiency for women/men and additionally for sex for the overall population.

Each increment of one SD in SBP TTR was associated with a reduction in the MACCE risk in the overall population and the women subgroup in the unadjusted model (hazard ratio (HR), 0.84; 95%CI, 0.78–0.91; *p* < 0.001, and HR, 0.83; 95% CI, 0.72–0.96; *p* = 0.013, respectively) and the adjusted model (adjusted HR, 0.89; 95% CI 0.82–0.97; *p* = 0.007, and adjusted HR, 0.85; 95% CI, 0.74–0.99; *p* = 0.039, respectively), but not in the male subgroup. Moreover, the relationship between SBP TTR increases and the risk of acute decompensated heart failure also exhibited significant sex differences (adjusted HR, 0.68; 95% CI, 0.51–0.92; *p* = 0.011 for women, and adjusted HR, 0.94; 95% CI, 0.78–1.13; *p* = 0.526 for men). Additional details are provided in Table 2 and **Supplementary Fig. 1**.

**Fig. 1.**
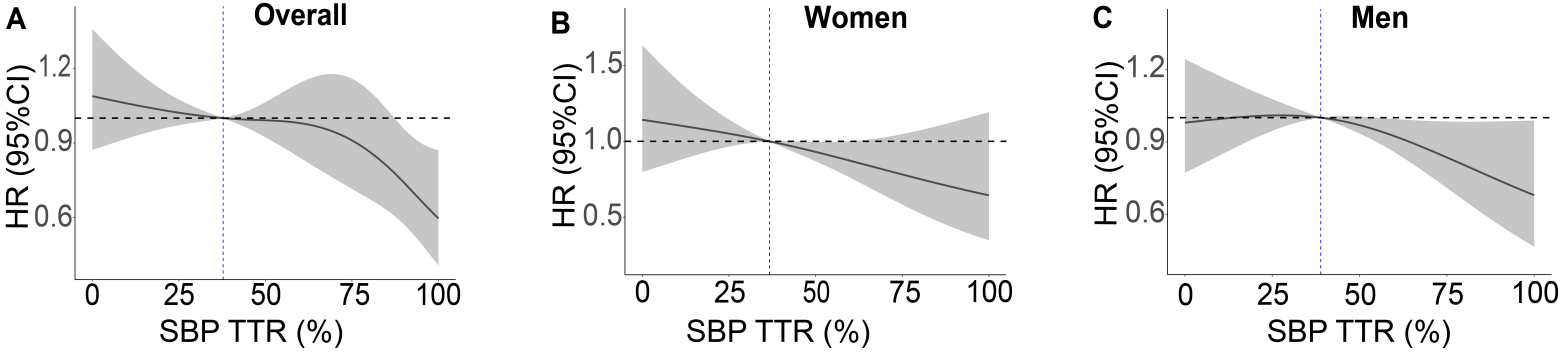
**Relationship between systolic blood pressure time in target range (SBP TTR) and major adverse cardiovascular and cerebrovascular events in (A) the overall population and by sex: (B) women; (C) men**. Restricted cubic splines were performed with the median SBP TTR as reference and the model was fully adjusted for age, race, treatment group, 10-year cardiovascular risk score, body mass index, renal insufficiency, and baseline SBP for women and men separately, with an additional adjustment for sex in the overall population.

Restricted cubic splines were utilized to investigate the relationship, with the median SBP TTR of the respective population as the reference. As depicted in Fig. 1, an elevation in SBP TTR was inversely correlated with the risk of MACCE for the overall population and the women subgroup. However, for men, an inverse relationship between SBP TTR and MACCE risk was clearly observed only when SBP TTR exceeded 39%. Similar outcomes were observed in the relationship between SBP TTR and acute decompensated heart failure (**Supplementary Fig. 2**).

**Fig. 2.**
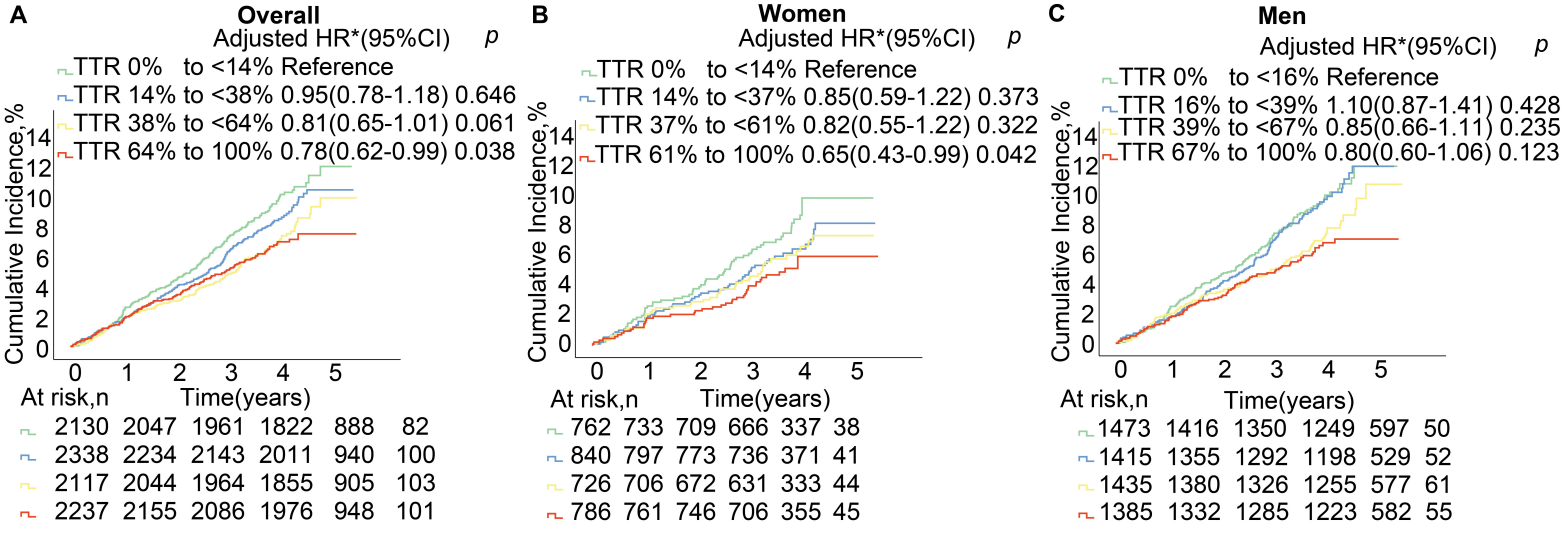
**Systolic blood pressure time in target range (SBP TTR) categories and risk of major adverse cardiovascular and cerebrovascular events in (A) the overall population and by sex: (B) women; (C) men**. Kaplan–Meier estimates and adjusted hazard ratios (adjusted HR; with 95% confidence intervals (CIs)) for age, race, treatment group, 10-year cardiovascular risk score, body mass index, renal insufficiency, and baseline SBP for women/men and additionally for sex for the overall population. To accurately reflect the impact of sex differences, our analysis utilized quartile groupings based on the SBP TTR for distinct populations: the overall population at 38% (14%–64%), women at 37% (14%–61%), and men at 39% (16%–67%).

To further explore this relationship, we stratified the participants via their respective SBP TTR quartiles: the overall population at 38% (14%–64%), women at 37% (14%–61%), and men at 39% (16%–67%). Elevated SBP TTR levels were correlative with a trend for reduced MACCE risk in the overall population and women, but in men, this trend was only observed when SBP TTR exceeded 39% (Fig. 2 and **Supplementary Table 5**).

### 3.3 Sensitivity Analysis

To further validate the sex-specific independent predictive role of SBP TTR on MACCE risk, we additionally adjusted for mean SBP or SBP variability in the adjusted model. After additional adjustment for mean SBP, a reduction in the risk of MACCE was observed with a one SD increment in SBP TTR in the overall population (adjusted HR 0.90; 95% CI, 0.82–0.98; *p* = 0.016) and women (adjusted HR 0.84; 95% CI, 0.72–0.98; *p* = 0.030), while for men, the association remained non-significant (adjusted HR 0.93; 95% CI, 0.84–1.04; *p* = 0.189). Similar sex differences were also seen in the risk for acute decompensated heart failure (adjusted HR, 0.85; 95% CI, 0.72–0.999; *p* = 0.049 for overall, adjusted HR, 0.64; 95% CI, 0.47–0.87; *p* = 0.004 for women, and adjusted HR, 0.97; 95% CI, 0.79–1.18; *p* = 0.732 for men). Similar results were noted after additional adjustment for SBP variability (**Supplementary Tables 6,7**).

## 4. Discussion

Our investigation has elucidated a sex-specific relationship between SBP TTR and cardiovascular event risk, indicating that an increment in SBP TTR is correlated with a reduced risk of MACCE and acute decompensated heart failure in the overall population and in women, while this relationship was not observed in men. In men, a similar trend was noted only when SBP TTR levels exceeded 39%. Furthermore, after additional adjustment for blood pressure variability and mean SBP, the sex-specific relationship between SBP TTR and cardiovascular event risk remained consistent.

### 4.1 Higher SBP TTR and Reduced Cardiovascular Risk

Hypertension serves as a key factor in the pathogenesis of atherosclerosis via increasing vascular shear stress, which leads to arterial wall thickening, development of atherosclerosis, plaque vulnerability, and ultimately an elevated risk of cardiovascular events [19, 20, 21, 22]. SBP TTR serves as an indicator of effective blood pressure control, accounting for levels, stability, and duration of blood pressure. Higher SBP TTR signifies lower and more consistent levels of vascular shear stress, leading to a reduction in arterial medial thickening and preventing tears and fragmentation in the internal elastic lamina [23]. Moreover, higher levels of SBP TTR diminish the mechanical stimulation of the vascular wall, decreasing the activation of intracellular signaling pathways triggered by mechanical factors and stabilizing cellular functions [24]. Eventually, patients with hypertension benefit from more effective blood pressure control which reduces the risk of cardiovascular diseases.

A previous study has demonstrated an inverse relationship between SBP TTR and the risk of MACCE and acute decompensated heart failure in patients with hypertension, and our study has yielded similar results [17]. Notably, when first investigating sex-specific relationships, the trend of reduced MACCE and acute decompensated heart failure risk with increasing SBP TTR was observed only when SBP TTR exceeded 39%. These findings underscore the importance of implementing sex-specific and personalized blood pressure management strategies to improve cardiovascular outcomes.

### 4.2 Sex-Specific Relationship Attributed to Sex Hormone Differences

Our study has elucidated a sex-specific relationship between SBP TTR and cardiovascular risk, which we hypothesize may be due to differences in sex hormones. Endogenous estrogen exerts cardioprotective effects by regulating nitric oxide (NO) biosynthesis and bioavailability, inhibiting the renin-angiotensin-aldosterone system (RAAS), modulating the sympathetic nervous system, and reducing endothelin-1 (ET-1) levels, thereby promoting vasodilation, inhibiting vascular remodeling, and decreasing arterial stiffness and cardiovascular risk [25, 26, 27, 28]. In postmenopausal women, estrogen deficiency also exacerbates endothelial dysfunction, promotes the rupture of arterial elastin, leads to collagen accumulation, and increases arterial stiffness [29]. In addition, estrogen deficiency is associated with the progression of metabolic syndrome, leading to adverse lipid distribution in elderly women [30]. Obesity, particularly a high BMI, is linked to adipose tissue dysfunction, elevated oxidative stress, activation of the RAAS system, and overactivity of the sympathetic nervous system, promoting chronic vascular inflammation and exacerbating cardiovascular risk [31]. These changes collectively result in greater arterial stiffness in postmenopausal women compared to age-matched men, with faster progression of vascular dysfunction, ultimately leading to higher cardiovascular risk.

In a large cross-sectional study involving 18,326 women, the proportion of postmenopausal women was approximately 90% in the 54–55 age group (n = 2324), 96% in the 56–57 age group (n = 2448), and 98% in the 58–59 age group (n = 2621) [32]. This indicates that the women in our study primarily belong to a postmenopausal population characterized by significantly reduced estrogen levels. These women also exhibited higher BMI levels compared to men, potentially due to adverse lipid distribution resulting from estrogen deficiency. The SBP TTR levels in women were also lower than those in men, despite using a consistent number of medications, indicating poorer blood pressure control in women. These findings suggest that the women in our study have a higher risk of hypertension and cardiovascular disease due to decreased estrogen levels. This presents greater challenges for optimizing blood pressure management and improving cardiovascular outcomes. Consequently, they may gain more significant benefits from effective blood pressure control reflected by increased SBP TTR, compared to men. In contrast, men only exhibit significant benefits after achieving higher SBP TTR levels (SBP TTR >39%).

The findings of this study underscore the importance of considering sex-specific differences in clinical blood pressure management strategies. Based on the findings of this study, we recommend implementing sex-specific management strategies for hypertensive patients in clinical practice. For female patients, particularly postmenopausal women, higher SBP TTR levels are strongly associated with reduced risks of MACCE and acute decompensated heart failure. Therefore, prioritizing SBP TTR stability at elevated levels is advised. Pharmacologic choices should favor agents proven to enhance blood pressure stability, with regular monitoring to ensure sustained SBP TTR over time. For male patients, while elevated SBP TTR also provides protective benefits, the clinical focus should be placed on maintaining higher thresholds (e.g., above 39%). Implementing sex-specific follow-up intervals and blood pressure monitoring strategies may enhance SBP TTR control, thereby improving cardiovascular outcomes.

### 4.3 Limitations

Our study has several limitations. First, the population was subject to rigorous blood pressure management, which limited fluctuations in blood pressure and converged the mean blood pressure and time in the target range. Second, the method for SBP TTR calculation is complex and the results are significantly affected by the selection of time periods for the analysis. Finally, this study is a post hoc analysis based on data from the SPRINT trial, a prospective randomized controlled trial that targeted a high cardiovascular risk population without a history of diabetes or stroke, which may have influenced the generalizability of the results to a large population.

## 5. Conclusions

In summary, there are significant sex-specific differences in the relationship between SBP TTR and the risk of cardiovascular events. In women, an elevation in SBP TTR is correlated with a reduced risk of MACCE and acute decompensated heart failure, while in men, a similar trend is observed only when SBP TTR reaches higher levels. These suggest that improving SBP TTR may be crucial in reducing cardiovascular event risk in patients with hypertension, and it is essential to consider sex differences and develop personalized blood pressure management strategies to optimize cardiovascular outcomes.

## Availability of Data and Materials

All datasets and materials relevant to this study have been made publicly accessible via the BioLINCC data retrieved at https://biolincc.nhlbi.nih.gov/home/. All SPRINT anonymized data can be found at the National Heart, Lung and Blood Institute (NHLBI) Biologic Specimen and Data Repository (https://biolincc.nhlbi.nih.gov/home/).

## Supplementary Material

Supplementary material associated with this article can be found, in the online version, at https://doi.org/10.31083/RCM26262.
