# Supplementary material for: Sex-Specific Association between Systolic Blood Pressure Time in Target Range and Cardiovascular Outcomes: A Post-Hoc Analysis of the SPRINT Trial
Source: Rev Cardiovasc Med. 2025 Mar 18;26(3):26262. doi: 10.31083/RCM26262 (PMC11951276; doi:10.31083/RCM26262)

**Supplementary Table S1. Baseline clinical characteristics by sex**

|  | **All (n=8822)** | **Women (n=3114)** | **Men (n= 5708)** | ***P* value** |
| --- | --- | --- | --- | --- |
| Intensive arms | 4421(50.1) | 1576(50.6) | 2845(49.8) | 0.491 |
| Age, years | 67.9±9.4 | 68.6±9.5 | 67.6±9.3 | <0.001 |
| BMI, kg/m^2^ | 29.8±5.6 | 30.1±6.5 | 29.7±5.1 | 0.007 |
| White | 5813(65.9) | 1743(56.0) | 4070(71.3) | <0.001 |
| Current smoking | 1135(12.9) | 395(12.7) | 740(13.0) | 0.708 |
| History of CVD | 1771(20.1) | 474(15.2) | 1297(22.7) | <0.001 |
| 10-y ASCVD risk | 17(16, 19) | 17(16-18) | 18(16-19) | <0.001 |
| Renal insufficiency^1^ | 2369(26.9) | 986(31.7) | 1383(24.2) | <0.001 |
| Baseline SBP, mmHg | 145.1±11.1 | 147.3±11.5 | 143.4±10.8 | <0.001 |
| Baseline DBP, mmHg | 80.2±11.5 | 79.9±11.9 | 80.4±11.3 | 0.052 |
| TTR, % | 38(14-64) | 37(14-61) | 39(16-67) | <0.001 |
| Mean SBP, mmHg | 129.8±12.1 | 129.5±13.0 | 129.8±11.5 | 0.241 |
| SBP variability, mmHg | 8.9±7.0 | 9.6±7.4 | 8.5±6.7 | <0.001 |

Data are presented as mean±SD, median (interquartile range), or n (%). ASCVD, arteriosclerotic cardiovascular disease; BMI, body mass index; CVD, cardiovascular disease; DBP, diastolic blood pressure; eGFR, glomerular filtration rate; SBP, systolic blood pressure; SD, standard deviation; TTR, time in target range.

^1^eGFR <60ml/min/1.73m^2^

**Supplementary Table S2. Antihypertensive agent by sex**

|  | **All（n=8822）** | **Women（n=3114）** | **Men**  **(n= 5708)** | ***P* value** |
| --- | --- | --- | --- | --- |
| **Number of agents** |  |  |  | 0.349 |
| 0 | 391(4.4) | 149(4.8) | 242(4.2) |  |
| 1 | 2457(27.9) | 838(26.9) | 1619(28.4) |  |
| 2 | 3535(40.1) | 1266(40.7) | 2269(39.8) |  |
| ≥3 | 2439(27.6) | 861(27.6) | 1578(27.6) |  |
| **Agent** |  |  |  |  |
| ACEI | 3868(43.8) | 1160(37.3) | 2708(47.4) | <0.001 |
| ARB | 1885(21.4) | 760(24.4) | 1125(19.7) | <0.001 |
| NS-BB | 36(0.4) | 11(0.4) | 25(0.4) | 0.551 |
| S-BB | 2950(33.4) | 1097(35.2) | 1853(32.5) | 0.009 |
| ND-CCB | 368(4.2) | 147(4.7) | 221(3.9) | 0.057 |
| D-CCB | 3068(34.8) | 1053(33.8) | 2015(35.3) | 0.161 |
| THZ | 4366(49.5) | 1645(52.8) | 2721(47.7) | <0.001 |
| LDIU | 342(3.9) | 130(4.2) | 212(3.7) | 0.284 |
| ALP | 285(3.2) | 25(0.8) | 260(4.6) | <0.001 |
| ALD | 127(1.4) | 65(2.1) | 62(1.1) | <0.001 |
| Other | 131(1.5) | 56(1.8) | 75(1.3) | 0.072 |

n (%). ACEI, angiotensin converting enzyme inhibitor; ARB, Angiotensin II Receptor Blocker; NS-BB, Non-Selective Beta-Blocker; S-BB, Selective Beta-Blocker; ND-CCB, non-dihydropyridine calcium channel blocker; D-CCB, dihydropyridine calcium channel blocker; THZ, thiazide diuretic; LDIU, loop diuretic; ALP, alpha-adrenergic antagonist; ALD, aldosterone receptor antagonist.

**Supplementary Table S3. Antihypertensive agent by sex and time in target range (TTR) categories**

|  | **TTR0% to <14%**  **(n=2130)** | | | **TTR14% to <38%**  **(n=2338)** | | | **TTR38% to <64%**  **(n=2117)** | | | **TTR64% to 100%**  **(n=2237)** | | | **Mean TTR, %** | | |
| --- | --- | --- | --- | --- | --- | --- | --- | --- | --- | --- | --- | --- | --- | --- | --- |
|  | **Women**  **(n=762)** | **Men**  **(n=1368)** | ***P***  **value** | **Women**  **(n=876)** | **Men**  **(n=1462)** | ***P***  **value** | **Women**  **(n=774)** | **Men**  **(n=1343)** | ***P***  **value** | **Women**  **(n=702)** | **Men**  **(n=1535)** | ***P***  **value** | **Women**  **(n=702)** | **Men**  **(n=1535)** | ***P***  **value** |
| **Number of agents** |  |  | 0.307 |  |  | 0.209 |  |  | 0.233 |  |  | 0.588 |  |  |  |
| 0 | 46(6.0) | 97(7.1) |  | 37(4.2) | 50(3.4) |  | 35(4.5) | 40(3.0) |  | 31(4.4) | 55(3.6) |  | 36±31 | 35±35 | 0.730 |
| 1 | 203(26.6) | 404(29.5) |  | 215(24.5) | 370(25.3) |  | 234 (30.2) | 405(30.2) |  | 186(26.5) | 440(28.7) |  | 40±30 | 42±32 | 0.054 |
| 2 | 285(37.4) | 492(36.0) |  | 387(44.2) | 598(40.9) |  | 292(37.7) | 541(40.3) |  | 302(43.0) | 638(41.6) |  | 40±29 | 43±31 | <0.001 |
| ≥3 | 228(29.9) | 375(27.4) |  | 237(27.1) | 444(30.4) |  | 213(27.5) | 357(26.6) |  | 183(26.1) | 402(26.2) |  | 38±30 | 41±30 | 0.016 |
| **Agent** |  |  |  |  |  |  |  |  |  |  |  |  |  |  |  |
| ACEI | 280(36.7) | 569(41.6) | 0.028 | 338(38.6) | 754(51.6) | <0.001 | 311(40.2) | 678(50.5) | <0.001 | 231(32.9) | 707(46.1) | <0.001 | 38±29 | 42±30 | <0.001 |
| ARB | 194(25.5) | 253(18.5) | <0.001 | 234(26.7) | 303(20.7) | <0.001 | 166(21.4) | 248(18.5) | 0.096 | 166(23.6) | 321(20.9) | 0.146 | 37±29 | 42±31 | <0.001 |
| NS-BB | 3(0.4) | 9(0.7) | 0.632 | 3(0.4) | 5(0.3) | >0.999 | 3(0.4) | 4(0.3) | >0.999 | 2(0.3) | 7(0.5) | 0.815 | 37±34 | 37±33 | 0.980 |
| S-BB | 267(35.0) | 456(33.3) | 0.425 | 318(36.3) | 505(34.5) | 0.389 | 254(32.8) | 421(31.3) | 0.485 | 258(36.8) | 471(30.7) | 0.004 | 39±30 | 41±31 | 0.146 |
| ND-CCB | 51(6.7) | 66(4.8) | 0.070 | 39(4.5) | 39(2.7) | 0.020 | 26(3.4) | 42(3.1) | 0.771 | 31(4.4) | 74(4.8) | 0.674 | 34±31 | 44±35 | 0.009 |
| D-CCB | 252(33.1) | 502(36.7) | 0.094 | 289(33.0) | 516(35.3) | 0.257 | 270(34.9) | 453(33.7) | 0.590 | 242(34.5) | 544(35.4) | 0.657 | 40±30 | 41±32 | 0.166 |
| THZ | 379(49.7) | 592(43.3) | 0.004 | 463(52.9) | 724(49.5) | 0.119 | 410(53.0) | 657(48.9) | 0.073 | 393(56.0) | 748(48.7) | 0.001 | 40±30 | 43±31 | 0.002 |
| LDIU | 40(5.2) | 49(3.6) | 0.065 | 30(3.4) | 60(4.2) | 0.326 | 35(4.5) | 50(3.7) | 0.367 | 25(3.6) | 51(3.3) | 0.772 | 36±31 | 40±29 | 0.293 |
| ALP | 6(0.8) | 60(4.4) | <0.001 | 7(0.8) | 74(5.1) | <0.001 | 7(0.9) | 56(4.2) | <0.001 | 5(0.7) | 70(4.6) | <0.001 | 37±30 | 42±30 | 0.470 |
| ALD | 12(1.6) | 11(0.8) | 0.099 | 19(2.2) | 17(1.2) | 0.056 | 18(2.3) | 19(1.4) | 0.124 | 16(2.3) | 15(1.0) | 0.015 | 43±30 | 43±28 | 0.879 |
| Other | 16(2.1) | 27(2.0) | 0.843 | 11(1.3) | 14(1.0) | 0.498 | 14(1.8) | 25(1.9) | 0.931 | 15(2.1) | 9(0.6) | <0.001 | 41±32 | 33±28 | 0.133 |

Data are presented as n (%). ACEI, angiotensin converting enzyme inhibitor; ARB, Angiotensin II Receptor Blocker; NS-BB, Non-Selective Beta-Blocker; S-BB, Selective Beta-Blocker; ND-CCB, non-dihydropyridine calcium channel blocker; D-CCB, dihydropyridine calcium channel blocker; THZ, thiazide diuretic; LDIU, loop diuretic; ALP, alpha-adrenergic antagonist; ALD, aldosterone receptor antagonist.

**Supplementary Table S4. Crude Number of all Events by sex and SBP time in target range (TTR) categories.**

|  |  | **Women (n=3114)** | | | | **Men (n= 5708)** | | | |
| --- | --- | --- | --- | --- | --- | --- | --- | --- | --- |
|  | **All**  **(n=8822)** | **TTR**  **0% to <14%**  **(n=762)** | **TTR**  **14% to <38%**  **(n=876)** | **TTR**  **38% to <64%**  **(n=774)** | **TTR**  **64% to 100%**  **(n=702)** | **TTR**  **0% to <14%**  **(n=1368)** | **TTR**  **14% to <38%**  **(n=1462)** | **TTR**  **38% to 64%**  **(n=1343)** | **TTR**  **64% to 100%**  **(n=1535)** |
| Major adverse cardiovascular and cerebrovascular events | 673(7.6) | 62(8.1) | 56(6.4) | 50(6.5) | 36(5.1) | 134(9.8) | 134(9.2) | 95(7.1) | 106(6.9) |
| Nonfatal myocardial infarction | 258(2.9) | 23(3.0) | 20(2.3) | 22(2.8) | 13(1.9) | 53(3.9) | 52(3.6) | 37(2.8) | 38(2.5) |
| Non-MI ACS | 83(0.9) | 3(0.4) | 5(0.6) | 3(0.4) | 5(0.7) | 14 (1.0) | 22(1.5) | 14(1.0) | 17(1.1) |
| Stroke | 159(1.8) | 23(3.0) | 14(1.6) | 13(1.7) | 9(1.3) | 34(2.5) | 32(2.3) | 15(1.1) | 18(1.2) |
| Acute decompensated heart failure | 198(2.2) | 23(3.0) | 17(1.9) | 14(1.8) | 6(0.9) | 39(2.9) | 35(2.4) | 32(2.4) | 32(2.1) |
| Cardiovascular death | 127(1.4) | 8(1.0) | 8(0.9) | 12(1.6) | 7(1.0) | 25(1.8) | 32(2.2) | 12(0.9) | 23(1.5) |

ACS, acute coronary syndrome; MI, myocardial infarction; SBP, systolic blood pressure.

**Supplementary Table S5. Association between SBP time in target range (TTR) categories and risk of cardiovascular events**

|  | **Overall** | | **Women** | | **Men** | |
| --- | --- | --- | --- | --- | --- | --- |
|  | **Adjusted**^1^  **HR (95%CI)** | ***P* value** | **Adjusted**^1^  **HR (95%CI)** | ***P* value** | **Adjusted**^1^  **HR (95%CI)** | ***P* value** |
| **Major adverse cardiovascular and cerebrovascular events** |  |  |  |  |  |  |
| TTR 0% to <14% | Reference |  | Reference |  | Reference |  |
| TTR 14% to <38% | 0.95(0.78-1.18) | 0.646 | 0.82(0.56-1.18) | 0.279 | 1.03(0.80-1.32) | 0.822 |
| TTR 38% to <64% | 0.81(0.65-1.01) | 0.061 | 0.83(0.56-1.22) | 0.343 | 0.79(0.60-1.05) | 0.101 |
| TTR 64% to 100% | 0.78(0.62-0.99) | 0.038 | 0.66(0.43-1.01) | 0.058 | 0.85(0.65-1.12) | 0.259 |
| **Nonfatal myocardial infarction** |  |  |  |  |  |  |
| TTR 0% to <14% | Reference |  | Reference |  | Reference |  |
| TTR 14% to <38% | 0.98(0.70-1.36) | 0.898 | 0.88(0.48-1.63) | 0.689 | 1.02(0.69-1.51) | 0.928 |
| TTR 38% to <64% | 0.90(0.63-1.29) | 0.568 | 1.17(0.63-2.16) | 0.618 | 0.78(0.51-1.21) | 0.273 |
| TTR 64% to 100% | 0.78(0.54-1.14) | 0.197 | 0.78(0.38-1.58) | 0.485 | 0.77(0.50-1.21) | 0.260 |
| **Non-MI ACS** |  |  |  |  |  |  |
| TTR 0% to <14% | Reference |  | Reference |  | Reference |  |
| TTR 14% to <38% | 1.50(0.81-2.80) | 0.201 | 1.52(0.36-6.47) | 0.574 | 1.51(0.76-3.01) | 0.244 |
| TTR 38% to <64% | 1.02(0.51-2.04) | 0.965 | 1.03(0.20-5.28) | 0.972 | 1.02(0.47-2.19) | 0.970 |
| TTR 64% to 100% | 1.23(0.63-2.41) | 0.544 | 1.91(0.43-8.45) | 0.396 | 1.13(0.53-2.40) | 0.757 |
| **Stroke** |  |  |  |  |  |  |
| TTR 0% to <14% | Reference |  | Reference |  | Reference |  |
| TTR 14% to <38% | 0.77(0.52-1.15) | 0.207 | 0.48(0.25-0.96) | 0.037 | 1.02(0.62-1.67) | 0.946 |
| TTR 38% to <64% | 0.51(0.32-0.82) | 0.006 | 0.50(0.24-1.01) | 0.054 | 0.51(0.27-0.96) | 0.038 |
| TTR 64% to 100% | 0.49(0.30-0.80) | 0.004 | 0.38(0.17-0.85) | 0.018 | 0.60(0.33-1.11) | 0.101 |
| **Acute decompensated heart failure** |  |  |  |  |  |  |
| TTR 0% to <14% | Reference |  | Reference |  | Reference |  |
| TTR 14% to <37% | 0.78 (0.53-1.13) | 0.190 | 0.64(0.34-1.22) | 0.177 | 0.87(0.55-1.39) | 0.561 |
| TTR 37% to 61% | 0.77(0.52-1.15) | 0.206 | 0.59(0.30-1.19) | 0.141 | 0.89(0.54-1.46) | 0.643 |
| TTR 61% to 100% | 0.65(0.43-0.996) | 0.048 | 0.29(0.12-0.73) | 0.008 | 0.87(0.53-1.44) | 0.591 |
| **Cardiovascular death** |  |  |  |  |  |  |
| TTR 0% to <14% | Reference |  | Reference |  | Reference |  |
| TTR 14% to <38% | 1.25(0.78-2.01) | 0.350 | 0.97(0.36-2.62) | 0.953 | 1.39(0.81-2.38) | 0.227 |
| TTR 38% to <64% | 0.87(0.50-1.50) | 0.617 | 1.72(0.67-4.39) | 0.256 | 0.59(0.29-1.20) | 0.147 |
| TTR 64% to 100% | 1.09(0.65-1.85) | 0.737 | 1.30(0.44-3.78) | 0.635 | 1.09(0.60-2.00) | 0.773 |

ACS, acute coronary syndrome; HR, hazard ratio; MI, myocardial infarction

^1^adjusted for age, race, treatment group, 10-year cardiovascular risk score, body mass index, renal insufficiency, and baseline SBP for women/men and additionally for sex for the overall population;

**Supplementary Table S6. Association between SBP time in target range (TTR) and risk of cardiovascular events with adjustment for mean systolic blood pressure**

|  | Adjusted^1^ plus mean systolic blood pressure  HR^2^(95%CI) *P* value | | | | | |
| --- | --- | --- | --- | --- | --- | --- |
|  | Overall | | Women | | Men | |
| Major adverse cardiovascular and cerebrovascular events | 0.90(0.82-0.98) | 0.016 | 0.84(0.72-0.98) | 0.030 | 0.93(0.84-1.04) | 0.189 |
| Nonfatal myocardial infarction | 0.89(0.77-1.03) | 0.115 | 0.89(0.69-1.15) | 0.355 | 0.89(0.75-1.06) | 0.191 |
| Non-MI ACS | 0.96(0.75-1.21) | 0.705 | 1.27(0.76-2.13) | 0.356 | 0.89(0.68-1.17) | 0.414 |
| Stroke | 0.77(0.63-0.93) | 0.006 | 0.70(0.52-0.96) | 0.024 | 0.82(0.64-1.06) | 0.127 |
| Acute decompensated heart failure | 0.85(0.72-0.999) | 0.049 | 0.64(0.47-0.87) | 0.004 | 0.97(0.79-1.18) | 0.732 |
| Cardiovascular death | 1.07(0.87-1.33) | 0.515 | 1.21(0.83-1.77) | 0.319 | 1.04(0.80-1.35) | 0.756 |

ACS, acute coronary syndrome; HR, hazard ratio; MI, myocardial infarction; SD, standard deviation;

^1^adjusted for age, race, treatment group, 10-year cardiovascular risk score, body mass index, renal insufficiency, and baseline SBP for women/men and additionally for sex for the overall population;

^2^HR per 1-SD increase in time in target range;

**Supplementary Table S7. Association between SBP time in target range (TTR) and risk of cardiovascular events with adjustment for systolic blood pressure variability**

|  | Adjusted^1^ plus systolic blood pressure variability  HR^2^(95%CI) *P* value | | | | | |
| --- | --- | --- | --- | --- | --- | --- |
|  | Overall | | Women | | Men | |
| Major adverse cardiovascular and cerebrovascular events | 0.89(0.82-0.97) | 0.010 | 0.85(0.74-0.99) | 0.037 | 0.92(0.83-1.02) | 0.110 |
| Nonfatal myocardial infarction | 0.91(0.80-1.04) | 0.180 | 0.90(0.71-1.14) | 0.364 | 0.92(0.78-1.08) | 0.310 |
| Non-MI ACS | 0.96(0.76-1.21) | 0.720 | 1.30(0.78-2.18) | 0.319 | 0.90(0.69-1.17) | 0.416 |
| Stroke | 0.73(0.61-0.88) | 0.001 | 0.69(0.51-0.93) | 0.013 | 0.77(0.61-0.98) | 0.033 |
| Acute decompensated heart failure | 0.86(0.73-1.01) | 0.058 | 0.69(0.51-0.92) | 0.011 | 0.95(0.79-1.15) | 0.618 |
| Cardiovascular death | 0.96(0.79-1.17) | 0.672 | 1.16(0.82-1.65) | 0.396 | 0.90(0.71-1.15) | 0.408 |

ACS, acute coronary syndrome; HR, hazard ratio; MI, myocardial infarction; SD, standard deviation;

^1^adjusted for age, race, treatment group, 10-year cardiovascular risk score, body mass index, renal insufficiency, and baseline SBP for women/men and additionally for sex for the overall population;

^2^HR per 1-SD increase in time in target range;

**Figure Legends**

**Supplementary Fig.S1.** **Sex-Specific Impact of SBP TTR on Cardiovascular Outcomes**

**
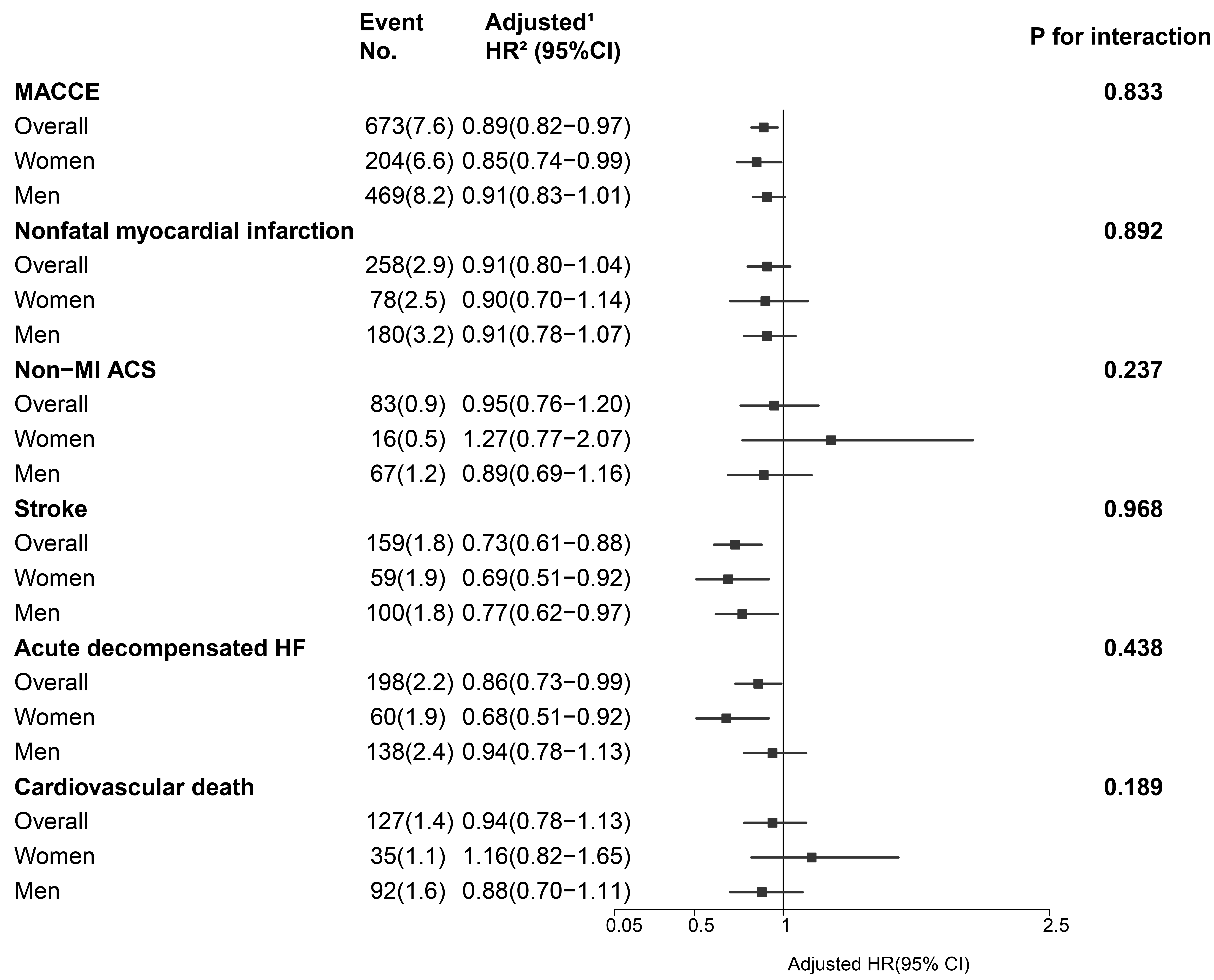
**

ACS, acute coronary syndrome; CI, confidence interval; HF, heart failure; HR, Hazard Ratio; MACCE, major adverse cardiovascular and cerebrovascular events; MI, myocardial infarction; No., number; SBP, systolic blood pressure; SD, standard deviation; TTR, time in target range;

^1^adjusted for age,sex, race, treatment group, 10-year cardiovascular risk score, baseline SBP, body mass index, and renal insufficiency;

^2^HR per 1-SD increase in time in target range.

**Supplementary Fig. S2.** Relationship between systolic blood pressure time in target range (SBP TTR) and acute decompensated heart failure in A the overall population and by sex: B women; C men.

Restricted cubic spines were performed with the median SBP TTR as reference and the model was adjusted hazard ratios (HR; with 95% confidence intervals) for age, race, treatment group, 10-year cardiovascular risk score, body mass index, renal insufficiency, and baseline SBP for women/men and additionally for sex for the overall population.


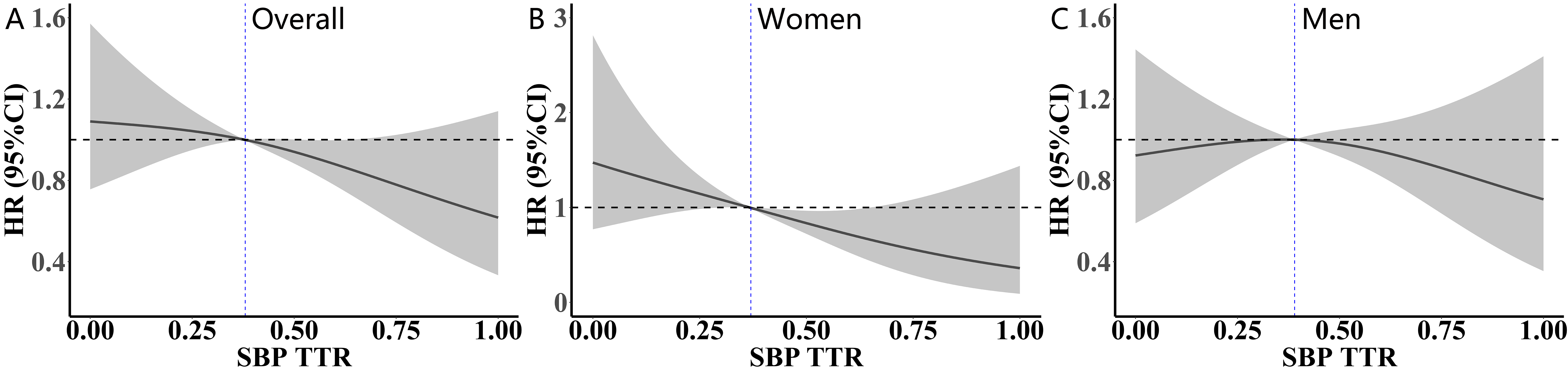

Supplement: Supplementary file 1 [file 2153-8174-26-3-26262-s1.docx]
